# Treatment outcomes and factors associated with unfavourable outcome among previously treated tuberculosis patients with isoniazid resistance in four regions of Cameroon

**DOI:** 10.11604/pamj.2020.37.45.25684

**Published:** 2020-09-10

**Authors:** Christopher Kuaban, Louise Daniele Ingrid Toukam, Melissa Sander

**Affiliations:** 1 Faculty of Health Sciences, The University of Bamenda, Bamenda Regional Hospital, Bamenda, Cameroon,; 2 Faculty of Health Sciences, The University of Bamenda, Bambili, Bamenda, Cameroon,; 3 Tuberculosis Reference Laboratory Bamenda, Bamenda, Cameroon

**Keywords:** Previously treated tuberculosis, isoniazid resistance, treatment outcome, Cameroon

## Abstract

**Introduction:**

it is unclear what the optimal treatment regimen for previously treated patients with rifampicin-susceptible isoniazid resistant tuberculosis should be. Conflicting evidence exists as to the effectiveness of the WHO standardized category II regimen in these patients. The objectives were to compare treatment outcomes between previously treated rifampicin-susceptible pulmonary tuberculosis patients with and without isoniazid resistance using the category II regimen and determine factors associated with an unfavourable outcome in those with isoniazid resistance in four regions of Cameroon.

**Methods:**

we conducted a retrospective review of all bacteriologically confirmed previously treated rifampicin-susceptible patients with and without isoniazid resistance registered in four regions of Cameroon from January 2012 to March 2015.

**Results:**

a total of 753 patients with a mean age of 38 ± 12 years including 498(66%) males were registered. Forty seven of the 753 had isoniazid-resistant TB, giving a prevalence of 6.2% (95% CI: 4.7-8.2). Treatment outcomes could only be ascertained for 733 patients as 20 (2.7%) were transferred out to other regions. Twenty-nine percent of patients with isoniazid resistance as against 21% of isoniazid susceptible patients had an unfavourable outcome (p = 0.32). In a multivariate logistic regression analysis, only HIV infection was significantly associated with an unfavourable outcome in isoniazid-resistant patients (p = 0.02).

**Conclusion:**

treatment outcomes using WHO category II regimen in previously treated rifampicin -susceptible pulmonary tuberculosis patients with and without isoniazid resistance in four regions of Cameroon are similar. HIV infection is an independent risk factor for an unfavourable outcome in patients with rifampicin-susceptible isoniazid-resistant disease treated with this regimen.

## Introduction

Tuberculosis (TB) is the first leading cause of death from a single infectious disease worldwide ranking above HIV/AIDS [1] despite the considerable efforts that have been made for its control. The latter is being hampered among other things especially in low income countries like Cameroon by difficult socio-economic conditions, the human immunodeficiency virus (HIV) infection and drug resistant TB particularly its multidrug resistant (MDR) form defined as TB resistant to at least rifampicin and isoniazid. These two drugs are the most inexpensive, the best tolerated and most effective drugs for the treatment of drug susceptible TB [2]. Studies worldwide have shown that previously treated TB patients have a much higher risk of harbouring MDR-TB bacilli than new patients [2]. As such many national tuberculosis control programmes therefore concentrate their efforts on these previously treated patients to detect cases of MDR-TB. Meanwhile it has also been shown that patients harbouring rifampicin-resistant TB strains have a similar bad prognosis as MDR-TB cases when treated with only first-line anti-TB drugs [3, 4]. For this reason, patients with rifampicin resistant TB are now treated like MDR-TB cases.

The availability of Xpert MTB/RIF assay (Cepheid Sunnyvale, CA, USA) which not only detects *Mycobacterium tuberculosis (M. tb)* in specimens but also its resistance to rifampicin has made it possible for previously treated TB patients to be screened rapidly for rifampicin resistance and be treated with the MDR-TB treatment regimen if this were to be the case. Patients who are not rifampicin resistant on the Xpert test are treated with the category II regimen comprising only first line anti-TB drugs without knowledge of whether their *M. tb* strains are resistant to isoniazid or not. The optimal treatment regimen for the management of rifampicin susceptible isoniazid resistant TB is not known. The question that can therefore be asked is whether the category II treatment regimen is adequate for the management of such patients although studies in the 1980´s reported a low rate of treatment failure in patients with isoniazid resistance receiving four or five first line drugs in 6 months regimens [5, 6]. This notwithstanding, some recent studies however reveal that patients with this form of TB disease have worse outcomes than those with susceptible disease [7-11] when treated with only first line drugs. The aim of this study was to determine the prevalence of isoniazid resistance among previously treated patients with rifampicin susceptible pulmonary tuberculosis (PTB) in four regions of Cameroon, compare treatment outcomes using the category II regimen between those with and without isoniazid resistance and determine factors associated with an unfavorable outcome in those with isoniazid resistance.

## Methods

Study setting: the study was conducted in all the TB diagnostic and treatment centres (DTCs) in four regions of Cameroon namely the North West, South West, West, and Littoral regions. In the DTCs of these regions and in accordance with the guidelines of the World Health Organization (WHO) [12], all previously treated PTB patients who present with symptoms suggestive of pulmonary tuberculosis, submit two sputum samples on two consecutive days for microscopic examination after staining by Ziel-Neelsen technique. Sputum specimens of all those who are smear positive for acid fast bacilli (AFB) on at least one of the sputum samples on microscopy are collected and sent to the Tuberculosis Reference Laboratory (TBRL) in Bamenda in the North West Region. In this laboratory, each patient´s sputum sample is first examined using the Xpert MTB/RIF assay. The results of this test are immediately communicated to the patients´ respective DTCs. Patients whose sputum samples show rifampicin resistance on the Xpert test are referred to one of the specialized MDR-TB treatment centres for management. Those whose tests reveal rifampicin susceptible TB are treated in their respective DTCs as retreatment cases with a category II regimen. The rest of the sputum sample received for each patient in the TBRL is cultured in liquid medium (BACTEC) and drug susceptibility testing to first and second line drugs of *M. tuberculosis* strains isolated from the cultures is carried out using the line probe assay (LPA-Genotype MTBDR plus version 2.0) and the indirect proportion method. Drugs normally tested in this laboratory include first line drugs (rifampicin, isoniazid, ethambutol) and second line drugs (quinolones and aminoglycosides).

All previously treated patients with rifampicin susceptible pulmonary tuberculosis are treated with a category II regimen for a total duration of 8 months. It consists of the daily administration of rifampicin I, isoniazid (H), ethambutol I, pyrazinamide (Z) and streptomycin (S) for the first two months, followed by the four drugs without streptomycin (intensive phase) for the next one month and then rifampicin, isoniazid and ethambutol for the last 5 months (2RHESZ/RHEZ/5RHE). Treatment in the intensive phase is administered under direct supervision of the health personnel while compliance to treatment during the continuation phase is assessed by monthly return for drug collection. During the 8 month treatment period each patient´s sputum sample is checked thrice for the presence or absence of AFB by direct microscopic examination. This is done at the end of the three month intensive phase and at the end of the fifth and eight month of treatment. In patients whose sputum smears remain positive at the end of the three month intensive phase of treatment, an additional month of intensive phase treatment is given followed by the continuation phase. The outcomes of patients at the end of the 8 months of treatment is recorded into one of the following six mutually exclusive categories, according to the recommendations of the World Health Organization [12]: 1) Cured: treatment completed with a negative sputum smear in the last month of treatment and on at least one previous occasion; 2) Treatment completed: patient who has completed treatment but does not meet the criteria to be classified as cure or failure; 3) Treatment failure: patient who is sputum smear positive at 5 months or later during treatment; 4) Died: patient who dies for any reason during the course of treatment. 5) Lost to follow-up: patient whose treatment is interrupted for two consecutive months or more; 6) Transferred-out: patient who has been transferred to another recording and reporting unit for whom treatment outcome is unknown.

Study design and population: the study population consisted of a retrospective cohort of all consecutive previously treated PTB patients who were put on a category II treatment regimen between January 2012 and May 2015 (duration of 39 months) in the DTCs of the four regions. Patients were included in the study if the TBRL had confirmed them as having bacteriologically confirmed rifampicin susceptible PTB and had carried out culture and drug susceptibility testing of *M. tuberculosis* strains isolated from their sputum samples. Patients with incomplete records, those lost to follow-up prior to commencement of treatment and those who were transferred to DTCs out of the four regions of the study were excluded.

Data collection: all previously treated PTB patients from the DTCs of the four regions whose sputum specimens were received in the TBRL in Bamenda during the study period for Xpert MTB/RIF assay as well as culture and drug susceptibility testing, were identified through a review of the laboratory´s register. For each patient identified, the following information was extracted from the register and recorded on a pre-prepared data collection form: names, age, sex, name of DTC and region, retreatment category of patient (relapse, treatment failure, and return after loss to follow-up), HIV status and anti-tuberculosis drug resistance profile of the patient. For patients whose *M. tuberculosis* strains showed resistance to isoniazid, the presence or absence of gene-mutations conferring isoniazid resistance (Kat G, InhA) determined by LPA was also extracted from the laboratory register and recorded. Using the patients socio-demographic characteristics obtained from the TBRL register, a review of the TB treatment registers and the patients´ treatment forms at their respective DTCs was carried out to extract and record information on the following: sputum smear results at baseline, at the end of the 3^rd^, 5^th^ and 8^th^ month of treatment as well as treatment outcomes (cured, treatment completed, treatment failure, death, loss to follow-up and transferred out). A patient was further classified as having a favorable outcome if he/she was cured or had completed treatment and as having an unfavorable outcome if he/she had treatment failure, died during the course of treatment or was lost to follow-up.

Data management and analysis: data were doubled entered into a computer using Epidata version 3.1 data entry software (Epidata Association, http:www.epidata dk) and discordances were identified and resolved through verification in the original paper record. Data analysis was performed using Epi-info version 7.2.2.6. Chi square or where appropriate, Fisher´s exact test was used to compare proportions while the student t-test was used to compare means. Multiple logistic regression analysis was performed using variables found to be significantly associated or having borderline association with unfavorable outcomes in the bivariate analysis to identify those that were independently associated with it. A p-value < 0.05 was used to characterize significant results.

Ethical and administrative approval: ethical clearance for the study was obtained from the Institutional Review Board of the Faculty of Health Sciences of the University of Bamenda. Administrative authorization to conduct the study in the DTCs and TBRL was granted by the National Tuberculosis Control Programme (NTCP) of Cameroon and the authorities of the TBRL.

## Results

**Figure 1:**
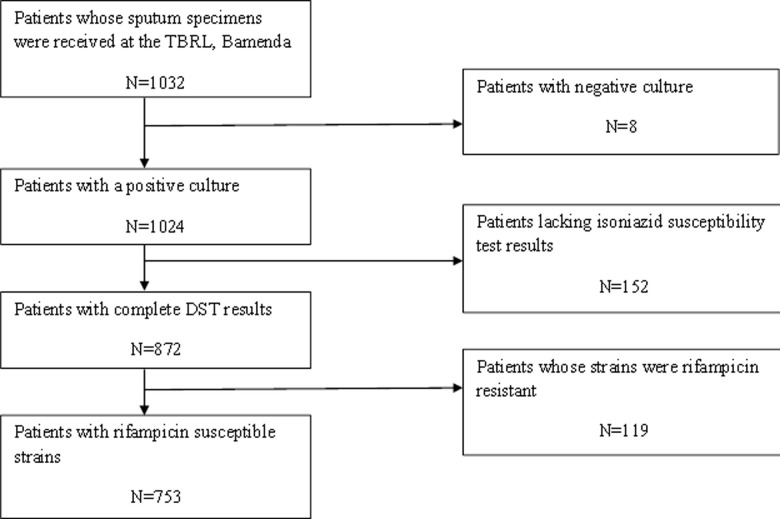
flow chart showing the selection of study participants

During the study period, sputum samples of 1032 previously treated PTB patients were received in the TBRL from all the DTCs of the four regions for Xpert MTB/RIF testing as well as for culture and drug susceptibility testing to first and second line anti-TB drugs. On a review of the laboratory results, of these 1032 patients, 279 (27%) were excluded from the study. The Figure 1 presents the flow chart showing the selection of patients retained for the study. There was however no statistical difference with respect to age and sex between those excluded and the 753 patients who were retained for the analysis.

Baseline characteristics of the study population: of the 753 participants included in the study, males made up the majority with a total of 498(66%). The age of the participants ranged from 9 to 84 years with a mean age of 38±12.5years. Sixty seven percent (n = 504) came from DTCs of the Littoral Region. The majority of the patients were relapses (71.1%) and 251 (33.3%) were HIV positive. Forty seven of the 753 patients included in the study harboured tubercle bacilli susceptible to rifampicin but resistant to isoniazid giving a rifampicin susceptible isoniazid resistance prevalence of 6.2% (95%CI: 4.7-8.2) (Table 1).

**Table 1:** general characteristics of study participants at baseline

Characteristics	Frequency (N=753)	Percentage (%)
**Gender**		
Male	498	66.1
Female	255	33.9
**Age group (years)**		
5-14	5	0.7
15-24	81	10.8
25-34	233	30.9
35-44	218	29.0
45-54	124	16.5
55-64	59	7.8
≥65	25	3.3
Unknown	08	1.1
**Patients´region of origin**		
Littoral	504	66..9
North West	116	15.4
West	108	14.3
South West	25	3.3
**Patients´ category with respect to previous TB treatment**		
Relapse	540	71.7
Return after default	171	22.7
Initial treatment failure	37	4.9
Other	5	0.7
**HIV status**		
Negative	443	58.8
Positive	251	33.3
Unknown	51	6.8
Resistance Profile		
Rifampicin and isoniazid susceptible	706	93.8
Rifampicin susceptible and isoniazid resistant	47	6.2

Outcomes of the rifampicin susceptible previously treated PTB patients with and without isoniazid resistance treated with a category II regimen over the study period: Table 2 compares outcomes of previously treated PTB patients with rifampicin susceptible disease with and without isoniazid resistance treated with a category II regimen over the study period. Excluding patients who were transferred out to DTCs out of the four regions studied (n = 20), the proportions of patients with isoniazid resistance with an unfavorable outcome (treatment failure, death and loss to follow-up) was 29% as against 21% for patients with isoniazid susceptible disease (P = 0.32).

**Table 2:** outcomes of rifampicin susceptible previously treated PTB patients with and without isoniazid resistance treated with the WHO category II regimen in four regions of Cameroon

Treatment Outcome	Previously treated TB patients			
	Rifampicin-Isoniazid susceptible		Rifamcipin susceptible isoniazid resistant	
	N=706	(%)	N=47	(%)
Cured	436	61.8	28	59.6
Treatment Completed	105	14.9	4	8.5
Died	56	7.9	7	14.9
Treatment failure	8	1.1	0	0.0
Loss to follow up	83	11.8	6	12.8
Transferred Out	18	2.5	2	4.3

Characteristics of the 47 previously treated PTB patients with rifampicin susceptible isoniazid resistant disease: the mean age of the 47 patients was 40±18.8 years (range 17-79 years) and 28(59.6%) of them were males. Of the 47 cases, 35(74.5%) came from DTCs of the Littoral Region while none came from the South West Region. Forty-one (87.2%) of the 47 patients were relapses and 20(42.6%) were HIV positive. The HIV status of three of the 47 patients was unknown. Drug susceptibility testing was determined for all the 47 patients by the indirect proportion method and in 40(85.1%) by line probe assay (LPA) as well. Of the 40 patients whose samples underwent drug susceptibility testing using LPA, 20(50%) had a Kat G mutation, 17(42.5%) an *InhA* mutation and 3(7.3%) had none of the mutations. A definitive treatment outcome was obtained for 45(95.7%) of the 47 patients as two were transferred out to DTCs outside the four study regions. Of these 45 patients 32(71.1%) had a favorable treatment outcome.

Factors associated with unfavorable treatment outcomes in previously treated rifampicin susceptible isoniazid resistant PTB patients: the characteristics of previously treated patients with rifampicin susceptible and isoniazid resistant PTB with favorable and unfavorable treatment outcomes are summarized in Table 3. Univariate analysis revealed that an unfavorable treatment outcome was associated with patients: 1) from the DTCs of the Littoral Region (P = 0.004); 2) who were HIV positive (P = 0.008); 3) whose tubercle bacilli presented Kat G mutations (P = 0.07) and *InhA* mutations (0.01). On the multivariate level including only these variables (Table 4), only the variable HIV positivity remained significantly associated with unfavorable treatment outcome (OR = 8.3; 95% CI; 1.3-53, P = 0.02).

**Table 3:** comparison of baseline characteristics of previously treated patients with rifampicin susceptible and isoniazid resistant PTB with favorable and unfavorable treatment outcomes

Characteristic	Treatment outcome		
	Favorable (N=32) %	Unfavorable (N=13) %	P Value
**Age group (years)**			
<45	71.9	61.5	0.49
≥45	28.1	38.5	
**Region of DTC**			
Littoral	81.3	53.9	0.004
North West	0	30.8	
West	18.7	15.3	
**Sex**			
Male	65.6	46.2	0.2
Female	34.3	53.8	
**HIV status**			
Positive	33.3	83.3	0.008
Negative	66.7	16.7	
**InhA Mutation**			
Yes	21.9	61.5	0.01
No	78.1	38.5	
**Kat G Mutation**			
Yes	53.1	23.1	0.07
No	46.9	76.9	

**Table 4:** factors associated with unfavorable treatment outcome of rifampicin-susceptible and isoniazid resistant PTB patients from 4 regions of Cameroon: multivariate analysis

Variable	β-coefficient	P-value	Odds ratio	95% CI
DTC of Littoral region	-1.7	0.2	0.3	0.1-1.7
HIV positivity	2.1	0.02	8.3	1.3-53
InhA mutations	0.8	0.4	2.3	0.2-18.5
Kat G mutations	-0.2	0.8	0.8	0.1-75

## Discussion

In this study, it was observed that only 6.2% of the previously treated PTB patients with culture confirmed tuberculosis harboured rifampicin-susceptible isoniazid-resistant tubercle bacilli. The prevalence of isoniazid resistance alone in previously treated PTB patients observed in our study is lower than the rate of 12% reported from a similar study in India [13]. It is equally lower than the 19.4% and 16.3% rates found among previously treated rifampicin susceptible PTB patients in Yaounde and the West Region of Cameroon respectively in 2000 [14, 15]. It is however similar to the 7.7% prevalence rate reported in previously treated patients by Meriki *et al*. [16] in previously from the North West and South West Regions of Cameroon in 2013. The high levels of isoniazid-resistant tuberculosis observed in the late 90s in Cameroon could be explained by the lack of a functional national TB control programme then. During that period patients were treated most often with regimens of drugs with infra-therapeutic doses as well as drugs of poor and /or unproven bioavailability and sometimes only with monotherapy [14]. The decrease in rates of resistance to isoniazid in Cameroonian previously treated TB patients as is currently observed could therefore simply be due to the implementation of a well-functioning TB control programme with drugs dispensed free of charge and administered under direct supervision especially during the intensive phase of treatment.

This study equally revealed a higher rate (29%) of an unfavorable treatment outcome (treatment failure, death, loss to follow-up) in patients with rifampicin-susceptible isoniazid-resistant PTB as compared to the group of patients (21%) with no isoniazid resistance. The difference was however not statistically significant (P = 0.32). The higher rate of an unfavorable outcome in the rifampicin-susceptible isoniazid-resistant group of patients in this study was probably due to the higher mortality rate observed in this group (Table 2). Our results are in line with those of other authors who equally reported treatment success rates in patients with rifampicin susceptible isoniazid resistant TB similar to those of patients with TB susceptible to both rifampicin and isoniazid [5, 6, 17, 18]. Our results are not in agreement however with those of other authors including a meta-analysis published in 2009 [9, 10, 13]. The difference in our results and these studies may be due to the fact that our patients received a daily administered category II treatment regimen (2RHEZS/RHEZ/5RHE) while these did not. For example in the study from India [13], the patients received this same regimen but it was administered thrice weekly (2R3H3E3Z3S3/R3H3E3Z3/5R3H3E3) and the authors recommended that the Indian National Tuberculosis Control Programme should consider transitioning from using an intermittent treatment regimen to daily dosing. Meanwhile in the study from Georgia [10], the patients received a nine month daily regimen of only three drugs: rifampicin, pyrazinamide and ethambutol.

Of the factors that showed a significant relationship with unfavorable treatment outcomes in previously treated rifampicin-susceptible isoniazid-resistant TB patients in this study on univariate analysis (Table 3), only a positive HIV status remained a significant independent determinant of an unfavourable outcome on multivariate analysis. In this study out of the 12 previously treated isoniazid resistant TB patients, with unfavorable outcomes, 10 were HIV infected. Four (40%) of these died while the remaining 6 were lost to follow up. Several possible reasons can be given to explain this association. Firstly, as has been reported by other authors [19], HIV positive patients who died in our cohort of patients may have been severely immune depressed with severe extensive lung or disseminated disease which could lead to death. Secondly, patients who were lost to follow up may have abandoned treatment because of the heavy pill burden they had to endure for treatment of their TB/HIV co-infection. Finally these patients could have abandoned treatment due to adverse effects of the combined use of anti-retroviral therapy and anti- tuberculosis drugs which as it is well known, can lead to severe adverse reactions [20]. Our results are not however in line with those of some authors [20, 21] who reported no association between HIV positive status and unfavorable treatment outcomes. The difference between our results and those of these authors may be due to the fact that patients in their studies still had high CD4+ cell counts and therefore still had a strong immune system. Unfortunately in our study, we did not dispose of CD4+ cell counts and chest X-rays for our patients to be able to assess the severity of both the HIV infection and the extent of lung involvement by the TB disease.

Our study was limited by the retrospective design and the operational nature of the study relying on records routinely maintained by the National Tuberculosis Control Programme (NTCP). However, we think that the supervision and monitoring system of the programme is robust and likely to have minimal impact on our results. Its strength lies in the fact that it is the first study to assess treatment outcomes and factors associated with unfavorable outcomes in previously treated tuberculosis patients with isoniazid resistance in Cameroon.

## Conclusion

The prevalence of isoniazid resistance in previously treated rifampicin susceptible pulmonary tuberculosis patients in four regions of Cameroon is relatively low. There is no significant difference in the treatment success rates in previously treated-rifampicin susceptible patients with and without isoniazid resistance treated with the WHO category II regimen. HIV infection is the most important predictor of an unfavorable treatment outcome in previously treated rifampicin-susceptible isoniazid-resistant tuberculosis patients from these regions. As such, previously treated rifampicin-susceptible isoniazid-resistant TB patients who are HIV infected should be closely monitored when on treatment in a bid to better manage adverse drug reactions as well as reduce loss to follow-up.

### What is known about this topic

- Optimal treatment regimen for previously treated patients with rifampicin-susceptible isoniazid-resistant tuberculosis not known;
- Conflicting evidence as to the effectiveness of the WHO category II treatment for the management of previously treated tuberculosis patients with rifampicin-susceptible isoniazid-resistant tuberculosis.

### What this study adds

- Treatment outcomes of previously treated rifampicin susceptible pulmonary tuberculosis patients with or without isoniazid resistance treated with the WHO category II regimen are similar;
- HIV infection is the only independent risk factor associated with an unfavourable outcome in patients with isoniazid-resistant disease.
